# Emicizumab is efficacious in people with hemophilia A with comorbidities aged ≥50 years: analysis of 4 phase III trials

**DOI:** 10.1016/j.rpth.2024.102405

**Published:** 2024-04-10

**Authors:** Víctor Jiménez-Yuste, Johannes Oldenburg, Eunice Tzeng, Elise Lim, Fabian Sanabria, Johnny Mahlangu

**Affiliations:** 1 Hematology Department, Hospital Universitario La Paz, IdiPAZ, Autónoma University, Madrid, Spain; 2 Institute of Experimental Haematology and Transfusion Medicine, University of Bonn, Bonn, Germany; 3 Genentech, Inc., South San Francisco, California, USA; 4 F. Hoffmann-La Roche Ltd, Basel, Switzerland; 5 Faculty of Health Sciences, University of the Witwatersrand and National Health Laboratory Service, Johannesburg, South Africa

**Keywords:** comorbidities, comorbidity, emicizumab, hemophilia A, prophylaxis

## Abstract

**Background:**

The treatment of older people with hemophilia A (HA) can be complicated by comorbidities.

**Objectives:**

This post hoc analysis evaluates the efficacy and safety of emicizumab in people with HA aged ≥50 years with cardiovascular (CV) risk factors or HIV and/or hepatitis C virus (HCV) infection.

**Methods:**

The HAVEN 1 (NCT02622321), HAVEN 3 (NCT02847637), HAVEN 4 (NCT03020160), and STASEY (NCT03191799) studies enrolled adults/adolescents with severe HA. Participants were categorized as having a comorbidity if they had any CV risk factors (including history of CV disease, hypertension, diabetes, hyperlipidemia, prior stroke, or obesity), HIV, and/or HCV infection. Efficacy and safety outcomes were compared by age (<50 vs ≥50 years).

**Results:**

Of 504 participants at data cutoff, 408 were aged <50 years and 96 were aged ≥50 years. In people with HA aged <50 years, 26.7% had ≥1 CV risk factor and 29.4% had HIV and/or HCV infection. In people with HA aged ≥50 years, 72.9% had ≥1 CV risk factor and 74.0% had HIV and/or HCV infection. The mean (95% CI) annualized bleed rate for treated bleeds was 1.29 (0.07-6.06) for people with HA aged <50 years and 1.82 (0.19-6.93) for people with HA aged ≥50 years. No significant differences in annualized bleed rates were observed for those with comorbidities compared with those without. Safety outcomes were similar regardless of age.

**Conclusion:**

This pooled analysis suggests that emicizumab efficacy and safety in people with HA aged ≥50 years with CV and HIV/HCV comorbidities were consistent with those in people with HA aged <50 years enrolled in the HAVEN 1, 3, and 4 and STASEY studies.

## Essentials

• Older people with hemophilia A may have other diseases at the same time (comorbidities).
• This analysis addresses the data gap of emicizumab in people with hemophilia A aged ≥50 years with comorbidities.
• Emicizumab efficacy and safety were not adversely affected by comorbidities seen in older people with hemophilia A.
• Outcomes in people with hemophilia A aged ≥50 years with comorbidities were similar to those in the population aged <50 years.

## 1 Introduction

Hemophilia A (HA) is an X-linked inherited bleeding disorder that results in impaired thrombin generation due to deficient factor (F)VIII activity [1]. The disease is characterized by frequent spontaneous and traumatic bleeding, primarily into joints but also into muscles and soft tissues [1]. The adoption of prophylaxis as the standard of care has resulted in improved life expectancy for people with HA, approaching that of the general population, and the introduction of innovative therapies in recent years has changed the clinical landscape of HA [2].

Age is an independent risk factor for cardiovascular (CV) events in the general population [3]. As people with HA reach more advanced ages, the management of their HA is increasingly complicated by the development of comorbid conditions and progression of chronic diseases, with limited formal guidance or published research available on the care of this population [2]. The increasing number of older people with HA presents new challenges, including the management of those with comorbidities, such as CV risk factors, CV disease, and hepatitis C virus (HCV) and HIV infection [2]. The safety and efficacy of standard-of-care therapies for HA should be evaluated in this challenging population of older people with HA with comorbidities, who have different clinical characteristics and risk factors from those of the overall population of people with HA and who may be receiving treatment for multiple conditions at once.

Emicizumab, a recombinant, humanized, bispecific monoclonal antibody, bridges activated FIX and FX, substituting for the cofactor function of deficient activated FVIII and improving hemostasis in people with HA [4,5]. Emicizumab has been evaluated and studied in people with HA of all ages and disease severities, with and without FVIII inhibitors. In the United States, it is indicated for routine prophylaxis in adult and pediatric people with HA, with or without FVIII inhibitors; however, the exact label varies by country [6]. More than 20,000 people have been treated with emicizumab globally to date [7], and the number continues to rise. Previously, the HAVEN 1, 3, and 4 studies and the STASEY study demonstrated the safety and efficacy of emicizumab in both adults and adolescents aged ≥12 years with severe HA with or without FVIII inhibitors [[8], [9], [10], [11]]. In addition, the HAVEN 2 study demonstrated efficacious bleed prevention and no new safety signals in children with HA with FVIII inhibitors [12]. Further to this, primary analysis of the HAVEN 6 study has shown efficacy and a favorable safety profile of emicizumab in people with moderate or mild HA [13]. A post hoc analysis of the HAVEN 1, 3, and 4 studies found that emicizumab trough concentrations were maintained in obese and nonobese people with HA, and annualized bleed rates (ABRs) for treated bleeds were comparable between the 2 groups [14]. However, there remains a data gap regarding the use of emicizumab in the treatment of older people with HA with comorbidities, where healthcare professionals may still face challenges, including managing the risk of thrombotic events (TEs) [15,16].

This post hoc analysis of people with severe HA in 4 phase III studies (HAVEN 1, 3, and 4 and STASEY) evaluates and compares the efficacy and safety of emicizumab in people with HA aged <50 years and people with HA aged ≥50 years with CV risk factors or HIV and/or HCV infection.

## 2 Methods

### 2.1 Study design and participants

This was a post hoc analysis of 4 phase III studies: HAVEN 1 (NCT02622321), 3 (NCT02847637), and 4 (NCT03020160) and STASEY (NCT03191799). All studies enrolled people with severe HA aged ≥12 years. HAVEN 1 and STASEY enrolled people with HA with FVIII inhibitors, while HAVEN 3 enrolled people with HA without FVIII inhibitors. HAVEN 4 enrolled people with HA regardless of FVIII inhibitor status.

People with HA were excluded from these studies if they had severe hepatic disease defined as total bilirubin >1.5 × the upper limit of normal (excluding Gilbert’s syndrome) and both aspartate transaminase and alanine transaminase >3 × the upper limit of normal at the time of screening, including history or known laboratory or radiographic evidence consistent with cirrhosis; HIV infection with a CD4 count <200 cells/μL; and concurrent disease, treatment, or abnormality that could impact safe participation (as deemed by the investigator).

All studies were conducted in accordance with the Declaration of Helsinki and International Conference on Harmonisation of Good Clinical Practice. The study protocols were approved by the relevant independent ethics committee/institutional review board at each participating institution, and all participants provided written informed consent.

In this post hoc analysis, people with HA aged ≥50 years were categorized as having a comorbidity if they had any of the following CV risk factors: past medical history (PMH) of CV disease or prior stroke; current evidence of hypertension, diabetes, hyperlipidemia, or obesity (defined as a body mass index [BMI] ≥30 kg/m^2^); HCV infection (defined as prior/current infection); or HIV infection. Family history of CV disease and smoking status were not included in this analysis due to inconsistent data collection. Participants were assessed for PMH of CV events prior to the initiation of emicizumab prophylaxis. Baseline joint arthropathy was defined as a participant having hemophilic arthropathy, arthropathy, or arthroplasty and joint procedures (ie, joint replacement, synovectomy, or synoviorthesis) in their medical history.

In each of the 4 phase III studies, emicizumab prophylaxis was administered subcutaneously, and participants received initial loading doses of emicizumab 3 mg per kilogram of body weight once a week for 4 weeks. Subsequently, maintenance doses of emicizumab were administered as follows: 1.5 mg/kg once weekly in HAVEN 1 and STASEY; 1.5 mg/kg once weekly or 3 mg/kg every 2 weeks in HAVEN 3; and 6 mg/kg every 4 weeks in HAVEN 4.

### 2.2 Objectives

Efficacy of emicizumab in people with HA with or without FVIII inhibitors aged <50 years or ≥50 years with CV risk factors or HIV and/or HCV infection was assessed and compared via evaluation of ABRs. Mean (95% CI) ABRs for all treated bleeds (defined as a bleed followed by treatment for a bleed), as well as treated joint and target joint bleeds were assessed. In addition, the adjusted ABR for treated bleeds was evaluated, in which *P* values were obtained via negative binomial regression, adjusting for baseline bleed levels and the effects of pooling participants from different studies. Bleeds were defined per the HAVEN 1, 3, and 4 and STASEY study protocols [[8], [9], [10], [11]].

The safety of emicizumab was assessed and compared between people with HA aged <50 years and those aged ≥50 years with CV risk factors or HIV and/or HCV infection. Key safety endpoints included incidence and severity of adverse events (AEs), serious AEs (SAEs), grade 3 to 4 AEs, local injection-site reactions (ISRs), and AEs of special interest (including TEs and thrombotic microangiopathies [TMAs]).

## 3 Results

### 3.1 Participant demographics and clinical characteristics

As of the clinical cutoff dates for each study (May 15, 2020, for HAVEN 1, 3, and 4; January 11, 2021, for STASEY), data were available for 504 people with severe HA across the 4 study populations (Table 1). The median (range) emicizumab treatment duration was 2.04 (0.02-4.25) years, and the median (range) age across all study participants was 33 (12-80) years. Across the 4 studies, 408 participants were <50 years of age and 96 were ≥50 years of age. From the overall pooled population of the 4 studies (*N* = 504), 35.5% of participants (*n* = 179) had ≥1 CV risk factor, and 9.7% (*n* = 49) had ≥2 CV risk factors. A total of 0.8% (*n* = 4) of participants had HIV infection only, 25.4% (*n* = 128) had HCV infection only, and 11.7% (*n* = 59) had HCV and HIV coinfection. Overall, most participants were White (65.7%; *n* = 331) and non-Hispanic (82.9%; *n* = 418; Supplementary Table S1).

**Table 1:** Baseline demographics, disease characteristics, and comorbidities.

Characteristic	Overall pooled population (*N* = 504)	People with HA aged <50 y (*n* = 408)	People with HA aged ≥50 y (*n* = 96)
Age (y), median (range)	33 (12-80)	28 (12-49)	57 (50-80)
Emicizumab treatment duration (y), median (range)	2.04 (0.02-4.25)	2.05 (0.02-4.20)	2.02 (0.14-4.25)
Participants with FVIII inhibitors, *n* (%)	283 (56.2)	234 (57.4)	49 (51.0)
No. of bleeds in 24 wk prior to study, median (range)^a^	7 (0-180)	7 (0-180)	6.5 (0-84)
Target joints at baseline, *n* (%)			
1 joint	102 (20.3)	86 (21.1)	16 (16.7)
≥1 joint	336 (66.8)	276 (67.8)	60 (62.5)
Arthropathy at baseline,^b^*n* (%)			
No	239 (47.4)	206 (50.5)	33 (34.4)
Yes	265 (52.6)	202 (49.5)	63 (65.6)
CV risk factors,^c^*n* (%)			
≥1 CV risk factor	179 (35.5)	109 (26.7)	70 (72.9)
≥2 CV risk factors	49 (9.7)	25 (6.1)	24 (25.0)
Hypertension	106 (21.0)	49 (12.0)	57 (59.4)
Hyperlipidemia	23 (4.6)	14 (3.4)	9 (9.4)
Diabetes	30 (6.0)	14 (3.4)	16 (16.7)
BMI ≥30 kg/m^2^	71 (14.1)	56 (13.7)	15 (15.6)
HIV and/or HCV infection,^d^*n* (%)			
HIV infection only	4 (0.8)	3 (0.7)	1 (1.0)
HCV infection only	128 (25.4)	80 (19.6)	48 (50.0)
HCV + HIV coinfection	59 (11.7)	37 (9.1)	22 (22.9)
PMH of CV event, *n* (%)	19 (3.8)	11 (2.7)	8 (8.3)

BMI, body mass index; CV, cardiovascular; FVIII, factor VIII; HA, hemophilia A; HCV, hepatitis C virus; PMH, prior medical history.

a Bleeds were defined per the HAVEN 1, 3, and 4 and STASEY study protocols.

b Baseline joint arthropathy was defined as a participant having hemophilic arthropathy, arthropathy, or arthroplasty and joint procedures (ie, joint replacement, synovectomy, or synoviorthesis) in their medical history.

c CV risk factors included PMH of CV disease or prior stroke or current evidence of hypertension, diabetes, hyperlipidemia, or obesity (BMI ≥30 kg/m^2^).

d HCV infection was defined as prior/current infection.

Joint arthropathy was present at baseline in 49.5% (*n* = 202) of participants <50 years of age; 26.7% (*n* = 109) had ≥1 CV risk factor, and 6.1% (*n* = 25) had ≥2 CV risk factors. A total of 0.7% (*n* = 3) of participants <50 years of age had HIV infection only, 19.6% (*n* = 80) had HCV infection only, and 9.1% (*n* = 37) had HCV and HIV coinfection. Arthropathy was present at baseline in 65.6% (*n* = 63) of participants aged ≥50 years, while 72.9% (*n* = 70) had ≥1 CV risk factor, and 25.0% (*n* = 24) had ≥2 CV risk factors (Table 1). Only 1 participant in this older population had HIV infection only; 50.0% (*n* = 48) of participants had HCV infection only, and 22.9% (*n* = 22) had HCV and HIV coinfection.

Across all comorbidity subgroups, the median emicizumab treatment duration was numerically similar (Table 2). Incidence of FVIII inhibitors was similar between participants with ≥1 and ≥2 CV risk factors (47.1% and 50.0%, respectively) and HCV-positive only participants (58.3%). A lower proportion of participants with HIV and HCV coinfection had inhibitors to FVIII (9.1%; Table 2).

**Table 2:** Baseline demographics and characteristics in people with hemophilia A aged ≥50 years by comorbidity subgroup.

	People with HA aged ≥50 y (*n* = 96)					
Characteristic	Total (*n* = 96)	≥1 CV risk factor (*n* = 70)	≥2 CV risk factors (*n* = 24)	HIV-positive only (*n* = 1)	HCV-positive only (*n* = 48)	HIV- + HCV-positive (*n* = 22)
Age (y), median (range)	57 (50-80)	58 (50-80)	61 (50-77)	50 (50-50)	56.5 (50-76)	53.5 (50-67)
Participants with FVIII inhibitors, *n* (%)	49 (51.0)	33 (47.1)	12 (50.0)	0 (0.0)	28 (58.3)	2 (9.1)
No. of bleeds in 24 wk prior to study, median (range)^a^	6.5 (0-84)	6.5 (0-84)	8.0 (0-35)	5.0 (5-5)	7.0 (0-63)	6.0 (0-49)
Emicizumab treatment duration (y), median (range)	2.02 (0.14-4.25)	2.02 (0.14-3.69)	1.98 (0.14-3.69)	2.96 (2.96-2.96)	2.07 (0.46-4.25)	1.92 (0.14-3.61)
Target joints at baseline, *n* (%)						
1 joint	16 (16.7)	12 (17.1)	4 (16.7)	1 (100.0)	9 (18.8)	1 (4.6)
≥1 joint	60 (62.5)	45 (64.3)	16 (66.7)	1 (100.0)	32 (66.7)	12 (54.6)
Arthropathy at baseline,^b^*n* (%)						
No	33 (34.4)	26 (37.1)	11 (45.8)	0	16 (33.3)	5 (22.7)
Yes	63 (65.6)	44 (62.9)	13 (54.2)	0	32 (66.7)	17 (77.3)

CV, cardiovascular; FVIII, factor VIII; HA, hemophilia A; HCV, hepatitis C virus.

a Bleeds were defined per the HAVEN 1, 3, and 4 and STASEY study protocols.

b Baseline joint arthropathy was defined as a participant having hemophilic arthropathy, arthropathy, or arthroplasty and joint procedures (ie, joint replacement, synovectomy, or synoviorthesis) in their medical history.

Among the pooled population, a total of 3.8% (*n* = 19) of participants had a PMH of a CV event (Table 1). There were 3 PMH events of myocardial infarction in 2 participants, 3 participants with coronary artery disease, and 1 participant with arteriosclerosis. Peripheral venous disease was present in 1 participant. There were 3 cases of thrombosis: 1 device-related thrombosis, 1 infection-induced thrombosis, and 1 subclavian vein thrombosis. Of the 19 participants with PMH of CV risk factors, 42.1% (*n* = 8) were ≥50 years of age, with 1 of these participants having a PMH of both coronary artery disease and myocardial infarction.

Details of concomitant medication in relation to CV risk factors or viral infection are given in Supplementary Table S2. While these data are based on concomitant medication records, participants did not keep an ongoing diary documenting such medication use during the trials.

### 3.2 Efficacy (ABRs for treated bleeds)

**Figure 1:**
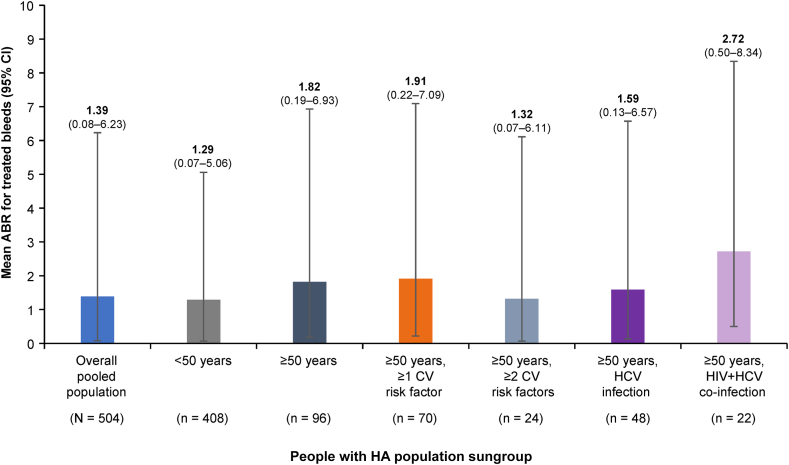
Mean annualized bleed rates (ABRs) for treated bleeds by age group and comorbidity. Ninety-five percent CI was derived from exact Poisson distribution. *N/n* represents the number of participants. Data from the one participant with HIV infection only have been omitted. CI, confidence interval; CV, cardiovascular; HCV, hepatitis C virus; People with HA, people with hemophilia A.

The mean (95% CI) ABR for treated bleeds for people with HA aged <50 years (*n* = 408) was 1.29 (0.07-6.06), while for those aged ≥50 years (*n* = 96), it was 1.82 (0.19-6.93) (Table 3). Mean (95% CI) ABR for treated bleeds was consistent across subgroups in people with HA aged ≥50 years with ≥1 CV risk factor (1.91 [0.22-7.09]), ≥2 CV risk factors (1.32 [0.07-6.11]), and HCV infection (1.59 [0.13-6.57]; Figure 1). Mean (95% CI) ABRs were 1.59 (0.13-6.56) in people with HA aged ≥50 years with hypertension and 2.08 (0.27-7.35) in those with a BMI ≥30 kg/m^2^. People with HA aged ≥50 years with HIV and HCV coinfection (*n* = 22) in particular had a numerically higher mean (95% CI) ABR for treated bleeds of 2.72 (0.50-8.34) compared with people with HA aged <50 years; however, this was not statistically significant (*P* = .459).

**Table 3:** Mean annualized bleed rates for treated bleeds for people with hemophilia A with or without factor VIII inhibitors by age group and comorbidity.

	Overall pooled population (*N* = 504)	People with HA aged <50 y (*n* = 408)	People with HA aged ≥50 y (*n* = 96)				
			Total (*n* = 96)	≥1 CV risk factor (*n* = 70)	≥2 CV risk factors (*n* = 24)	HCV-positive only (*n* = 48)	HIV- + HCV-positive (*n* = 22)
All participants							
Mean ABR for treated bleeds (95% CI)	1.39 (0.08-6.23)	1.29 (0.07-6.06)	1.82 (0.19-6.93)	1.91 (0.22-7.09)	1.32 (0.07-6.11)	1.59 (0.13-6.57)	2.72 (0.50-8.34)
Participants with FVIII inhibitors							
*n* (%)	283 (56.2)	234 (57.4)	49 (51.0)	33 (47.1)	12 (50.0)	28 (58.3)	2 (9.1)
Mean ABR for treated bleeds (95% CI)	1.41 (0.09-6.27)	1.45 (0.10-6.34)	1.22 (0.05-5.95)	1.15 (0.04-5.84)	0.55 (0.00-4.77)	1.63 (0.14-6.63)	2.65 (0.48-8.24)
Participants without FVIII inhibitors							
*n* (%)	221 (43.8)	174 (42.6)	47 (49.0)	37 (52.9)	12 (50.0)	20 (41.7)	20 (90.9)
Mean ABR for treated bleeds (95% CI)	1.36 (0.08-6.18)	1.07 (0.03-5.69)	2.44 (0.39-7.91)	2.59 (0.45-8.15)	2.08 (0.27-7.35)	1.54 (0.12-6.48)	2.72 (0.5-8.35)

Data from the one participant with HIV infection only have been omitted.

ABR, annualized bleed rate; CI, confidence interval; CV, cardiovascular; FVIII, factor VIII; HA, hemophilia A; HCV, hepatitis C virus.

**Figure 2:**
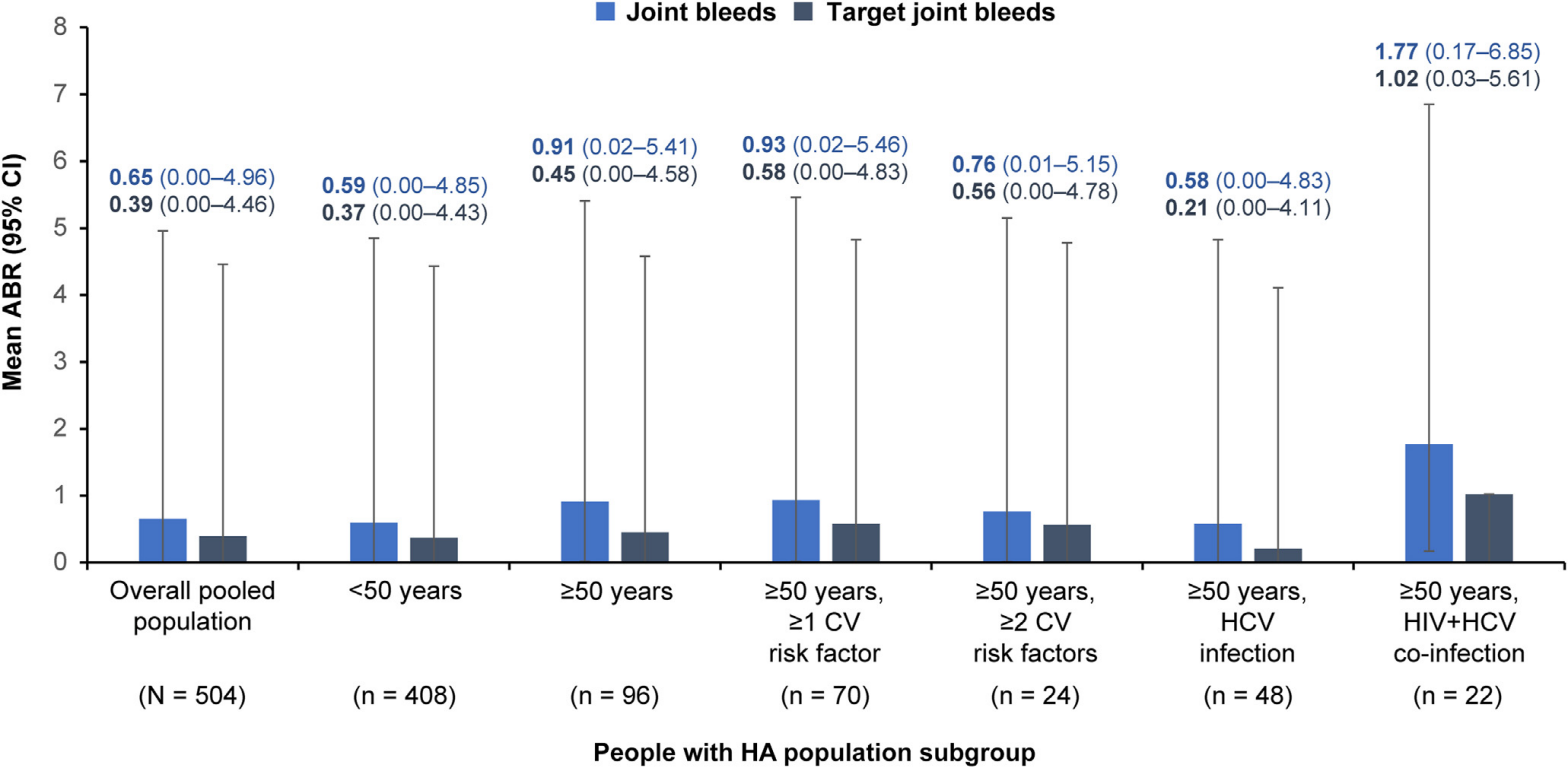
Mean annualized bleed rates (ABRs) for treated joint and target joint bleeds by age group and comorbidity. Data from the one participant with HIV infection only have been omitted. CI, confidence interval; CV, cardiovascular; HCV, hepatitis C virus; People with HA, people with hemophilia A.

ABRs for treated joint and target joint bleeds were consistent across people with HA aged <50 years and those aged ≥50 years with comorbidities (Figure 2). For participants aged <50 years, mean (95% CI) ABRs for treated bleeds were numerically higher in participants with FVIII inhibitors (1.45 [0.10-6.34]) compared with those without inhibitors (1.07 [0.03-5.69]; Table 3). For participants aged ≥50 years, mean (95% CI) ABRs for treated bleeds were numerically lower in participants with FVIII inhibitors (1.22 [0.05-5.95]) compared with those without inhibitors (2.44 [0.39-7.91]; Table 3). For participants aged ≥50 years with CV risk factors, mean ABRs were higher in those without FVIII inhibitors, while there was no notable difference for the groups with HCV only or HCV and HIV co-infection. For the group of people with HA aged ≥50 years, no significant differences in ABRs were observed for those with CV risk factors, HIV infection, or HCV infection compared with those without these comorbidities (Table 4).

**Table 4:** Adjusted annualized bleeding rates for treated bleeds in people with hemophilia A aged ≥50 years with comorbidities versus those without comorbidities.

	People with HA with comorbidity		People with HA without comorbidity		Rate ratio (95% CI)	*P* value^a^
Comorbidity	*n*	Lsmean ABR (95% CI)	*n*	Lsmean ABR (95% CI)		
≥1 CV risk factor	70	1.48 (0.91-2.40)	26	2.28 (1.00-5.23)	0.65 (0.25-1.68)	.37
≥2 CV risk factors	24	1.07 (0.46-2.52)	72	1.85 (1.16-2.97)	0.58 (0.22-1.51)	.26
HCV infection only	48	1.71 (0.92-3.16)	48	1.63 (0.90-2.92)	1.05 (0.45-2.48)	.91
HCV or HIV	71	1.78 (1.11-2.85)	25	1.27 (0.53-3.03)	1.41 (0.53-3.71)	.49
HCV and HIV coinfection	22	2.20 (0.92-5.28)	74	1.47 (0.87-2.47)	1.50 (0.51-4.42)	.46

Data from the one participant with HIV infection only have been omitted.

ABR, annualized bleed rate; CI, confidence interval; CV, cardiovascular; HA, hemophilia A; HCV, hepatitis C virus; Lsmean, least-squares mean.

a *P* values were obtained via negative binomial regression, adjusting for the effects of study and baseline bleed levels.

### 3.3 Safety

AEs, SAEs, grade 3 to 4 AEs, and ISR rates were similar among people with HA aged <50 years and those aged ≥50 years (Table 5). Among people with HA aged <50 years, 91.4% (*n* = 373) had ≥1 AE and 22.1% (*n* = 90) had an ISR. TEs and TMAs occurred in 1.0% (*n* = 4) and 0.7% (*n* = 3) of participants aged <50 years, respectively.

**Table 5:** Safety summary.

Outcome, *n* (%)	Overall pooled population (*N* = 504)	People with HA aged <50 y (*n* = 408)	People with HA aged ≥50 y (*n* = 96)				
			Total (*n* = 96)	≥1 CV risk factor (*n* = 70)	≥2 CV risk factors (*n* = 24)	HCV-positive only (*n* = 48)	HIV- + HCV-positive (*n* = 22)
Any AE	462 (91.7)	373 (91.4)	89 (92.7)	66 (94.3)	22 (91.7)	45 (93.8)	21 (95.5)
SAE	97 (19.2)	74 (18.1)	23 (24.0)	16 (22.9)	2 (8.3)	11 (22.9)	5 (22.7)
Grade 3-4 AE	107 (21.2)	82 (20.1)	25 (26.0)	18 (25.7)	5 (20.8)	13 (27.1)	4 (18.2)
Local ISR	102 (20.2)	90 (22.1)	12 (12.5)	9 (12.9)	3 (12.5)	2 (4.2)	6 (27.3)
TE	6 (1.2)	4 (1.0)	2 (2.1)	2 (2.9)	0	1 (2.1)	0
TMA	3 (0.6)	3 (0.7)	0	0	0	0	0

Data from the one participant with HIV infection only have been omitted.

AE, adverse event; CV, cardiovascular; HA, hemophilia A; HCV, hepatitis C virus; ISR, injection-site reaction; SAE, serious adverse event; TE, thrombotic event; TMA, thrombotic microangiopathy.

Among people with HA aged ≥50 years, 92.7% (*n* = 89) had ≥1 AE and 12.5% (*n* = 12) had an ISR. Across comorbidity subgroups, the proportion of participants with ≥1 AE ranged from 91.7% to 95.5%. One TE each occurred in 2 participants who both had a CV risk factor, one of whom also had HCV infection. No TMAs were observed in the older population.

## 4 Discussion

In this pooled analysis from the HAVEN 1, 3, and 4 and STASEY clinical studies, emicizumab prophylaxis had a consistent efficacy and safety profile across participants aged <50 and ≥50 years with common comorbidities including CV risk factors or HIV and/or HCV infection. There were no significant differences in ABRs between those with comorbidities and those without. This analysis represents one of the largest datasets to examine treatment efficacy and safety among older people with hemophilia and comorbidities. Across several real-world studies investigating the efficacy and safety of emicizumab prophylaxis in people with HA with comorbidities, only 1 analysis was conducted in participants aged ≥50 years, highlighting the importance of this analysis in contributing to the data gap in the population of aging people with HA [17].

Presence and early onset of CV risk factors, including hypertension, are common among people with HA [[18], [19], [20]]. The increasing prevalence of obesity in people with hemophilia both increases the risk of CV disease and has an impact on bleeding, presenting a rising challenge in hemophilia care [21]. There are varied findings on the relationship between HA and CV disease. Some studies have shown reduced CV morbidity in people with HA, indicating that HA may lessen the impact of CV disease [15,20,22]; however, others have found greater or similar rates of CV morbidity and risk factors in people with HA compared with those in the general population [18,19,[23], [24], [25], [26]]. Background rates of such risk factors are not well established, particularly in an aging HA population within this new disease and treatment landscape [27]. As a result, additional research is needed to understand CV disease in this subgroup. Irrespective of these varied findings, people with HA with CV comorbidities are a distinct population and are challenging to treat [15].

HIV infection is associated with increased risk of heart disease [28]. Additionally, coinfection of HIV with HCV increases the risk of end-stage liver disease and acute and chronic renal disease, and worsens chronic HCV progression [29,30]. Infection by HIV, HCV, or hepatitis B virus remains the leading cause of death in people with HA, highlighting the need to understand this population [31]. Analysis of available data on mortality and causes of death (1968-2018) showed that the most frequently observed causes of death in people with HA were HIV, HCV, or hepatitis B virus infection (32.4%), followed by hemorrhage (21.4%); thromboses and CV-related deaths were less frequently reported [31]. Although new HIV and HCV infections are less of an issue in the modern management of people with HA, it remains relevant for older people with HA who were infected in the 1980s [32,33].

While 21.0% (*n* = 106/504) of the overall population in this study had hypertension, this rose to 59.4% (*n* = 57/96) when considering only the participants aged ≥50 years. This is in agreement with data reported in previous studies on people with HA [18,34], and is similar to the rate estimated for the general population of people without hemophilia of the same age [35]. The proportion of participants with obesity was also high but did not vary greatly between those aged <50 (13.7%; *n* = 56) and ≥50 years (15.6%; *n* = 15), perhaps reflecting a general tendency for people with HA to limit their physical activity to reduce the risk of bleeding.

The numbers of bleeds in the 24 weeks prior to study enrollment were similar among participants aged <50 years and ≥50 years and were generally comparable across those with ≥1 and ≥2 CV risk factors and with HIV, HCV, or HIV and HCV coinfection. Target joints at baseline were also similar between these groups. As might be expected, the proportion of participants with hemophilic arthropathy at baseline was slightly lower in participants <50 years of age than in those ≥50 years of age with CV risk factors and HIV, HCV, or HIV and HCV coinfection. People with HA aged ≥50 years are less likely to have benefited from early prophylaxis compared with people with HA <50 years of age, which contributes to the higher occurrence of hemophilic arthropathy and joint damage [33], possibly accounting for the numerically higher ABRs observed. The higher rates of arthropathy may also have led to increased joint pain in the group ≥50 years of age. For some participants, this joint pain may have been erroneously interpreted as bleeding and subsequently treated at home, which may have also contributed to the higher ABR in this group compared with the group <50 years of age. Despite the difference in baseline joint arthropathy between the 2 age groups, the joint and target joint ABRs remained similar for participants <50 years and ≥50 years of age. Further, pooled data from long-term follow-up on participants from all ages enrolled in HAVEN 1 to 4 showed 95.1% (504/530) of target joints had been resolved over 120.4 weeks of emicizumab treatment [36]. Similarly, in the STASEY study final analysis, 93.8% of participants experienced zero target joint bleeds [37]. The phase IIIb/IV SPINART trial showed that after 3 years of follow-up, participants with established arthropathy on FVIII prophylaxis experienced a 94% reduction in number of bleeds; however, magnetic resonance imaging–assessed hemophilic arthropathy remained unchanged over time [38].

Of note, the proportion of participants with baseline joint arthropathy was lower in the current study than expected, especially in participants ≥50 years of age. An explanation for this may lie in the collection of baseline arthropathy data, which was based on medical records and targeted physical joint examination in the current study, rather than definitive determination of arthropathy via magnetic resonance imaging and ultrasound imaging [38,39]. As a result, there is a possibility that baseline joint arthropathy was underidentified.

ABRs for people with HA aged ≥50 years with CV risk factors such as hypertension and BMI ≥30 kg/m^2^ or with HIV, HCV, or HIV and HCV coinfection were similar to those seen for participants aged <50 years. ABR findings in this analysis are consistent with overall observations from a longitudinal observational study of people with HA aged ≥50 years [17]. In that study, median ABRs significantly decreased in people with HA with multiple CV risk factors and HIV infection after a median follow-up of 400 days on emicizumab prophylaxis. There were no significant differences between high-risk and standard-risk CV participants and no significant difference between participants with and without HIV infection [17].

Safety outcomes for the participants aged ≥50 years were also similar to those for the population aged <50 years and for the overall study population, indicating that emicizumab prophylaxis was well tolerated in these older people with HA with comorbidities. There was a slightly lower proportion of ISRs reported in people with HA ≥50 years of age (12.5%; *n* = 12) than in people with HA <50 years of age (22.1%; *n* = 90); as pain in the injection site was also reported as an ISR, it is likely that older participants were more accustomed to receiving injections. Few participants experienced a TE or TMA: 1.0% (*n* = 4) and 0.7% (*n* = 3), respectively, in people with HA <50 years of age, and 2.1% (*n* = 2) and 0.0%, respectively, in people with HA ≥50 years of age. Therefore, no meaningful comparison can be made between the different age groups and comorbidities regarding these AEs of special interest.

Safety outcomes from this post hoc analysis were also consistent with other studies in people with HA with comorbidities [17,40]. A separate analysis of emicizumab prophylaxis safety reports (cutoff May 2022) showed that 41 of 49 TEs not associated with activated prothrombin complex concentrate use were associated with ≥1 CV risk factor [40]. Real-world analysis of emicizumab prophylaxis in 17 people with HA ≥50 years of age with comorbidities demonstrated no TEs, TMAs, or reports of SAEs [17].

### 4.1 Limitations

Although the pooled population of these 4 clinical studies was quite large, the number of participants included in the analysis was relatively small compared with previous real-world studies [18,19,23,26]. There is a potential for bias, which may limit the applicability of this subgroup analysis, despite its concordance with other real-world studies of emicizumab [17]. Of note, only 1 participant ≥50 years of age had HIV infection alone, severely limiting the evaluation of this subgroup.

The 4 studies included in the analysis were not set up to specifically evaluate the impact of comorbidities on efficacy or safety. In addition, the protocols of the 4 studies specifically excluded those with a concurrent disease that would impact safe participation, as well as participants with HIV and a low CD4 count, which may have impacted analysis outcomes. Moreover, a lack of baseline data regarding rates and incidence of comorbid conditions in this analysis presented difficulties in judging the potential impact of treatments.

## 5 Conclusions

As the older population of people with HA provides a new challenge in the HA treatment landscape, there is a paucity of data on the management of older people with HA with comorbidities.

Despite the relatively small number of participants aged ≥50 years, this pooled analysis suggests that the efficacy and safety of emicizumab prophylaxis are not adversely affected by older age or common comorbidities seen in older people with HA. Outcomes in older people with HA with CV risk factors and HIV/HCV comorbidities were consistent with those of both younger people with HA and the overall population of people with HA enrolled in the HAVEN 1, 3, and 4 and STASEY studies.

This analysis highlights, however, that there remain a limited understanding and a need for published literature addressing the background rates of comorbid conditions in people with HA, as well as the optimal use of hemophilia therapies in this population.
