# Supplementary material for: Emicizumab is efficacious in people with hemophilia A with comorbidities aged ≥50 years: analysis of 4 phase III trials
Source: Res Pract Thromb Haemost. 2024 Apr 10;8(3):102405. doi: 10.1016/j.rpth.2024.102405 (PMC11112372; doi:10.1016/j.rpth.2024.102405)
Supplement: Supplementary material [file mmc1.docx]

**Supplementary material**

**Supplementary Table 1.** Comorbidities by race and ethnicity

|  | | | **Age ≥50 years** | | | | | | | | | | |
| --- | --- | --- | --- | --- | --- | --- | --- | --- | --- | --- | --- | --- | --- |
|  | | |  | **Cardiovascular comorbidities** | | | | | | | **HIV/HCV** | | |
| **Statistics** | **Overall population** | **Total aged ≥50 years** | | **PMH*** | **Hyper- tension** | **Hyper- lipidemia** | **Diabetes** | **BMI ≥30 kg/m^2^** | **≥1 CV risk factor** | **≥2 CV risk factors** | **HCV only** | **HIV only** | **HCV and HIV** |
| **Total participants, n** | 504 | 96 | | 8 | 57 | 9 | 16 | 15 | 70 | 24 | 48 | 1 | 22 |
| **Race, n (%)** | | | | | | | | | | | | | |
| American Indian or Alaska Native | 20 (4.0) | 1 (1.0) | | 0 | 0 | 0 | 0 | 0 | 0 | 0 | 0 | 0 | 0 |
| Asian | 100 (19.8) | 15 (15.6) | | 1 (12.5) | 9 (15.8) | 2 (22.2) | 4 (25.0) | 1 (6.7) | 11 (15.7) | 4 (16.7) | 9 (18.8) | 0 | 3 (13.6) |
| Black or African American | 27 (5.4) | 2 (2.1) | | 1 (12.5) | 2 (3.5) | 1 (11.1) | 1 (6.3) | 1 (6.7) | 2 (2.9) | 1 (4.2) | 2 (4.2) | 0 | 0 |
| Native Hawaiian or Other Pacific Islander | 3 (0.6) | 0 | | 0 | 0 | 0 | 0 | 0 | 0 | 0 | 0 | 0 | 0 |
| White, Hispanic or Latino in Ethnicity | 32 (6.3) | 2 (2.1) | | 0 | 1 (1.8) | 0 | 0 | 0 | 1 (1.4) | 0 | 0 | 1 (100) | 0 |
| White, Not Hispanic or Latino in Ethnicity | 292 (57.9) | 76 (79.2) | | 6 (75.0) | 45 (78.9) | 6 (66.7) | 11 (68.8) | 13 (86.7) | 56 (80.0) | 19 (79.2) | 37 (77.1) | 0 | 19 (86.4) |
| White, Not reported or Unknown in Ethnicity | 7 (1.4) | 0 | | 0 | 0 | 0 | 0 | 0 | 0 | 0 | 0 | 0 | 0 |
| Unknown | 23 (4.6) | 0 | | 0 | 0 | 0 | 0 | 0 | 0 | 0 | 0 | 0 | 0 |
| **Ethnicity, n (%)** | | | | | | | | | | | | | |
| Hispanic or Latino | 73 (14.5) | 3 (3.1) | | 0 | 1 (1.8) | 0 | 0 | 0 | 1 (1.4) | 0 | 0 | 1 (100) | 0 |
| Not Hispanic or Latino | 418 (82.9) | 92 (95.8) | | 8 (100) | 55 (96.5) | 9 (100) | 16 (100) | 15 (100) | 68 (97.1) | 24 (100) | 47 (97.9) | 0 | 22 (100) |
| Not reported or Unknown | 13 (2.6) | 1 (1.0) | | 0 | 1 (1.8) | 0 | 0 | 0 | 1 (1.4) | 0 | 1 (2.1) | 0 | 0 |

*CV disease, including acute myocardial infarction, aortic dilatation, arteriosclerosis, atrioventricular block, bundle branch block left, cardiac disorder, coronary artery disease, device-related thrombosis, hypertensive heart disease, and peripheral venous disease.

Abbreviations: BMI, body mass index; CV, cardiovascular; HCV, hepatitis C virus; HIV, human immunodeficiency virus; PMH, prior medical history.

**Supplementary Table 2.** Concomitant medications pertaining to CV risk factors and viral infection by comorbidity

|  | | **Aged ≥50 years** | | | | | | | | | | |
| --- | --- | --- | --- | --- | --- | --- | --- | --- | --- | --- | --- | --- |
|  | |  | **Cardiovascular comorbidities** | | | | | | | **HIV/HCV** | | |
|  | **Overall population** | **Total aged ≥50 years** | **PMH*** | **Hyper- tension** | **Hyper- lipidemia** | **Diabetes** | **BMI ≥30 kg/m^2^** | **≥1 CV risk factor** | **≥2 CV risk factors** | **HCV only** | **HIV only** | **HCV and HIV** |
| **Total participants, n** | 504 | 96 | 8 | 57 | 9 | 16 | 15 | 70 | 24 | 48 | 1 | 22 |
| **≥1 treatment in ATC Class Level 1, n (%)** |  |  |  |  |  |  |  |  |  |  |  |  |
| Anti-obesity preparations, excl. diet products | 10 (2.0) | 5 (5.2) | 1 (12.5) | 3 (5.3) | 1 (11.1) | 3 (18.8) | 2 (13.3) | 4 (5.7) | 3 (12.5) | 1 (2.1) | 0 | 2 (9.1) |
| Drugs used in diabetes | 15 (3.0) | 6 (6.3) | 1 (12.5) | 4 (7.0) | 2 (22.2) | 6 (37.5) | 1 (6.7) | 6 (8.6) | 4 (16.7) | 1 (2.1) | 0 | 3 (13.6) |
| Antivirals for systemic use^†^ | 115 (22.8) | 37 (38.5) | 4 (50.0) | 17 (29.8) | 4 (44.4) | 7 (43.8) | 4 (26.7) | 24 (34.3) | 7 (29.2) | 13 (27.1) | 1 (100) | 20 (90.9) |
| CV system | 234 (46.4) | 72 (75.0) | 5 (62.5) | 57 (100) | 9 (100) | 13 (81.3) | 14 (93.3) | 63 (90.0) | 24 (100) | 40 (83.3) | 1 (100) | 13 (59.1) |
| Agents acting on the renin-angiotensin system | 89 (17.7) | 50 (52.1) | 4 (50.0) | 48 (84.2) | 7 (77.8) | 9 (56.3) | 10 (66.7) | 49 (70.0) | 18 (75.0) | 27 (56.3) | 1 (100) | 10 (45.5) |
| Antihypertensives | 16 (3.2) | 2 (2.1) | 0 | 2 (3.5) | 0 | 0 | 0 | 2 (2.9) | 0 | 1 (2.1) | 0 | 0 |
| Beta-blocking agents | 45 (8.9) | 26 (27.1) | 3 (37.5) | 23 (40.4) | 4 (44.4) | 4 (25.0) | 5 (33.3) | 24 (34.3) | 10 (41.7) | 17 (35.4) | 0 | 4 (18.2) |
| Calcium channel blockers | 14 (2.8) | 5 (5.2) | 0 | 5 (8.8) | 0 | 1 (6.3) | 1 (6.7) | 5 (7.1) | 2 (8.3) | 2 (4.2) | 0 | 0 |
| Cardiac therapy | 72 (14.3) | 20 (20.8) | 1 (12.5) | 13 (22.8) | 3 (33.3) | 4 (25.0) | 3 (20.0) | 14 (20.0) | 7 (29.2) | 9 (18.8) | 0 | 6 (27.3) |
| Diuretics | 18 (3.6) | 9 (9.4) | 1 (12.5) | 8 (14.0) | 2 (22.2) | 1 (6.3) | 2 (13.3) | 8 (11.4) | 4 (16.7) | 6 (12.5) | 0 | 1 (4.5) |
| Lipid-modifying agents | 15 (3.0) | 8 (8.3) | 0 | 6 (10.5) | 1 (11.1) | 2 (12.5) | 0 | 6 (8.6) | 2 (8.3) | 4 (8.3) | 0 | 2 (9.1) |
| Peripheral vasodilators | 3 (0.6) | 0 | 0 | 0 | 0 | 0 | 0 | 0 | 0 | 0 | 0 | 0 |
| Vasoprotectives | 103 (20.4) | 22 (22.9) | 1 (12.5) | 15 (26.3) | 3 (33.3) | 4 (25.0) | 3 (20.0) | 17 (24.3) | 6 (25.0) | 11 (22.9) | 0 | 5 (22.7) |

Details of concomitant medications were collected at study enrolment. Participants did not keep a diary of the medications used during the course of the study; therefore, these data have limitations and may not be applicable to the comorbidities that were included in this analysis.
*PMH of CV disease, including acute myocardial infarction, aortic dilatation, arteriosclerosis, atrioventricular block, bundle branch block left, cardiac disorder, coronary artery disease, device-related thrombosis, hypertensive heart disease, and peripheral venous disease.

^†^All antiviral agents, including agents used for HIV and HCV as well as other viral conditions.

Abbreviations: ATC, anatomical therapeutic chemical; BMI, body mass index; CV, cardiovascular; HCV, hepatitis C virus; HIV, human immunodeficiency virus; PMH, prior medical history.
